# Cardiovascular Disease in Women and the Role of Hormone Replacement Therapy

**DOI:** 10.7759/cureus.69752

**Published:** 2024-09-19

**Authors:** Jomar N Machuca, Claudia P Rosales-Alvarez

**Affiliations:** 1 Internal Medicine, Veterans Affairs Medical Center, San Juan, PRI; 2 Cardiology, Veterans Affairs Medical Center, San Juan, PRI

**Keywords:** atherosclerosis, cardiovascular disease (cvd), coronary artery atherosclerosis, estrogen replacement therapy, ischemic heart disease (ihd), menopause, midlife women's health, traditional cardiovascular risk factors, vasomotor symptoms, women’s health

## Abstract

Cardiovascular disease (CVD) is the leading cause of death in women and manifests more severely and at a later stage in life compared to men. The low estrogen levels during menopause are linked to an increased CVD risk. This association has promoted research regarding the role of hormone replacement therapy (HRT) in reducing the symptoms and diseases related to menopause, including CVD. This review article aims to discuss the biological changes associated with menopause and their impact on CVD. It also examines the effects of HRT in women with comorbidities related to CVD, its indications, risks, and contraindications.

## Introduction and background

The mortality and prevalence of cardiovascular disease (CVD) are a significant concern, especially for women. In general, one person in the United States dies of CVD every half minute and of a stroke approximately every three minutes [1]. According to statistical data from the American Heart Association, almost half of women aged 20 and older have a cardiovascular condition, and over half of deaths from high blood pressure occur in women [2]. Surprisingly, less than half of women are aware that CVD is their leading cause of death [3]. These figures are so alarming that even the popular Time magazine highlighted in its cover story of a 2003 issue that one in three women will die from CVD [4].

Risk factors for CVD

In terms of risk factors for CVD in women of different ethnic groups, obesity is the most prevalent, particularly in White, African-American, and Hispanic women. In contrast, Asian women have a lower prevalence of obesity. Hypertension is the second most prevalent risk factor, followed by hypercholesterolemia and diabetes [3]. While the prevalence of these risk factors is similar in men and women, studies show that CVD is more severe in women [5]. In a study of more than 300,000 patients with myocardial infarction, mortality in women was significantly higher compared to men. Additionally, a higher proportion of women had myocardial infarction with non-obstructed coronary arteries (MINOCA) compared to men. Overall, mortality in women was much higher than in men, regardless of whether their coronary arteries were obstructed or not [3].

Ischemic heart disease in women 

Women tend to present MINOCA more frequently than men. This phenomenon has been linked to various cardiac manifestations, including microvascular dysfunction, vasospasm, and spontaneous coronary artery dissection, more commonly observed in women who had complications during pregnancy, such as preeclampsia [3]. The biological mechanisms contributing to these differences in CVD between men and women are complex. For instance, statistical analyses show that the incidence of coronary artery disease and cardiac infarction is delayed in women by 10 and 20 years, respectively, compared to men [2,5]. This delayed onset is related to the cardioprotective effects of estrogen during a woman's lifetime [5]. This association has prompted the development of several research studies to understand the mechanisms behind the hormone replacement therapy (HRT) effect on cardiovascular health.

## Review

There is a notable difference in the number of CVD-related events between premenopausal and postmenopausal women. The incidence of CVD tends to be higher in postmenopausal women compared to premenopausal women, a correlation attributed to the lower estrogen levels typically observed in postmenopausal women [5]. To better understand why this phenomenon occurs, it is essential to define the role of female reproductive hormones.

Female reproductive hormones 

Four types of estrogen have been described, including estrone, estradiol, estriol, and estetrol, which are named according to their amount of hydroxyl groups. For instance, estradiol has two hydroxyl groups and is the most potent and abundant, particularly in the female reproductive stage [6,7]. Estradiol plays a crucial role in the development of female reproductive organs and promotes endometrial thickening during the menstrual cycle. It also exerts specific effects on the vasculature. For example, it mediates nitric oxide synthase activation, resulting in transient vasodilation [8]. Estrogen also promotes vasodilation by modulating the renin-angiotensin system. This has been linked to less heart remodeling in various cardiac conditions, including ischemic heart disease [9]. In addition, estradiol inhibits smooth muscle proliferation and limits coronary artery disease progression through variations in gene expression [8,9]. On the other hand, the role of progesterone needs to be more well-defined.

**Figure 1:**
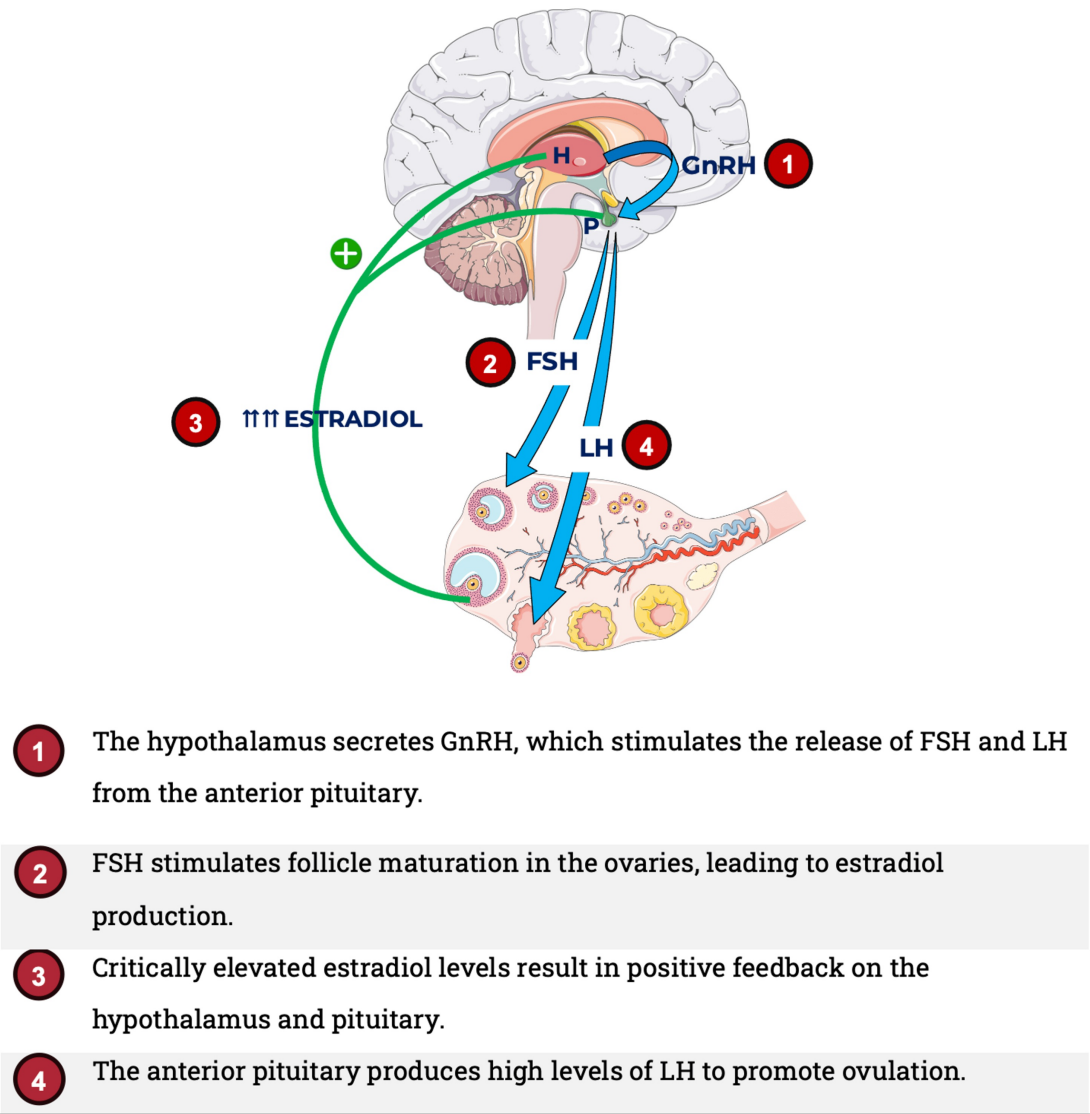
The ovulatory cycle H, hypothalamus; P, pituitary; GnRH, gonadotropin-releasing hormone; FSH, follicle stimulating hormone; LH, luteinizing hormone Source: This image was created by Dr. Jomar Machuca.

The hypothalamic-pituitary-gonadal axis involves a series of hormonal interactions that regulate the reproductive system (Figure 1). The pituitary gland releases follicle-stimulating hormone (FSH) to promote follicle development and estrogen production. Additionally, luteinizing hormone (LH) stimulates ovulation [10,11]. As a woman ages, her ovulatory cycles and follicle atrophy lead to decreased estrogen production [12]. The loss of estrogen-mediated negative feedback results in rising FSH levels as menopause approaches [11]. This leads to irregular menstrual periods and ultimately to the permanent cessation of menstruation for at least 12 consecutive months, defined as menopause [12].

Biological changes during menopause

Menopause typically occurs around the age of 52. More than 63 million women in the United States are 50 or older, and approximately 6,000 women reach menopause each day [13]. During menopause, women experience various symptoms, with the most common being vasomotor symptoms such as hot flashes, night sweats, and palpitations. Additionally, women may experience genitourinary syndrome, characterized by vulvovaginal atrophy, leading to dyspareunia and recurrent urinary tract infections [2,12,13]. It is important to note that while menopause itself does not cause CVD, it is a period where there is a significant increase in cardiovascular risk [2].

Vasculature

Unlike vasomotor symptoms, which tend to decrease over time, symptoms of genitourinary syndrome tend to increase [13]. Interestingly, vasomotor symptoms are associated with the development of cardiovascular events. Studies have shown a correlation between vasomotor symptoms and a nearly 30% increase in the risk of CVD [14]. They are also linked to adverse lipid profiles, insulin resistance, and hypertension. The menopausal transition is also associated with adverse vascular remodeling. For example, carotid intima-media thickness (cIMT), a measure of atherosclerotic disease, is accelerated in perimenopause regardless of a woman's age. Similarly, carotid-femoral pulse wave velocity, representing arterial stiffness, accelerates one year after the onset of menopause, irrespective of other risk factors [14].

Lipids

Regarding lipids, total cholesterol levels, especially low-density lipoprotein (LDL) and apolipoprotein B, increase around the onset of menopause [14]. Also, high-density lipoprotein (HDL) composition and distribution change, affecting retrograde cholesterol transport. In fact, higher levels of HDL during menopause are associated with an increased incidence of carotid atherosclerosis [2,14]. Additionally, during menopause, fat distribution shifts toward the visceral area, which is characteristic of metabolic syndrome and is related to increased cIMT [14].

Insulin Resistance 

The impact of menopause itself on insulin resistance and diabetes mellitus is still uncertain. However, it has been established that the hormonal changes during menopause can lead to insulin resistance [5]. This can have severe implications for women's health, as diabetes mellitus is linked to a fourfold increase in the risk of CVD in women. Diabetic women also face a poorer prognosis after myocardial infarction and an increased risk of heart failure [13]. Women with diabetes mellitus have a two to three times higher risk of mortality from CVD, cancer, and overall mortality, regardless of HRT treatment. [13] In view of these biological changes, many studies have been conducted to investigate the effect of HRT on menopausal symptoms and related diseases. 

History of HRT

Over the years, the use of HRT has had its highs and lows. Initially, HRT was thought to be beneficial for preserving youth and was used for the treatment of menopause symptoms. The first commercial estrogen product, extracted from the urine of pregnant women, was introduced in 1930. This was followed by the introduction of conjugated equine estrogen in 1942, which was used to treat vasomotor symptoms in postmenopausal women. Hormone prescriptions saw a significant increase in the 1960s after the publication of Feminine Forever, which portrayed menopause as a state of hormonal deficiency leading to a loss of sex appeal [13]. However, concerns about the risks of estrogen monotherapy, such as the increased risk of endometrial cancer, led to a decline in HRT use by 1965. The recognition of progesterone's role in reducing endometrial cancer risk in the 1980s resulted in a resurgence in HRT prescriptions. The Nurses’ Health Study further supported HRT use by demonstrating decreased cardiovascular and other mortalities, resulting in over 90 million HRT prescriptions annually [2,3,13]. In 2002, the Women's Health Initiative (WHI) study was published. It is the largest placebo-controlled randomized clinical trial to date that evaluated the risks and benefits of systemic HRT in the primary prevention of chronic diseases, including CVD. This study found increased risks of breast cancer, CVD, stroke, and thromboembolic events associated with HRT, leading to a significant decline in HRT prescriptions [13].

Observational Studies vs. Clinical Trials 

A discrepancy between observational studies and randomized clinical trials was noticed after the WHI study. Observational studies consistently showed reduced CVD with HRT, while clinical trials showed no effect. This discrepancy was attributed to the differences in the characteristics of women in both study types [5]. When comparing the female population in observational studies and clinical trials, it is important to note that the populations were different. In observational studies, women were relatively young with a normal BMI, while in randomized studies, they were generally older, had experienced menopause more than 10 years ago, and were overweight (Table 1) [5]. In essence, HRT had a positive effect on younger women who had recently experienced menopause, suggesting a critical window during which HRT can be beneficial. This phenomenon is described by the Timing Hypothesis, which proposes that the effects of HRT in preventing atherosclerosis depend on when the treatment is started in relation to the patient's age and the onset of menopause [5,11-14].

**Table 1:** Differences between participants in observation studies and clinical trials HRT, hormone replacement therapy Source: Hodis HN, Mack WJ: Menopausal hormone replacement therapy and reduction of all-cause mortality and cardiovascular disease: it’s about time and timing. The Cancer Journal. 2022, 28:208-23. 10.1097/ppo.0000000000000591

	Observational studies	Clinical trials
Age at the beginning of the study	30-55 years	63 years
Time at which HRT was started since the onset of menopause	<2 years	>10 years
Duration of HRT	>10 years	<7 years
Body mass index	25.1 kg/m^2^	28.5 kg/m^2^
Menopause symptoms	Predominant	Excluded

The Timing Hypothesis

**Figure 2:**
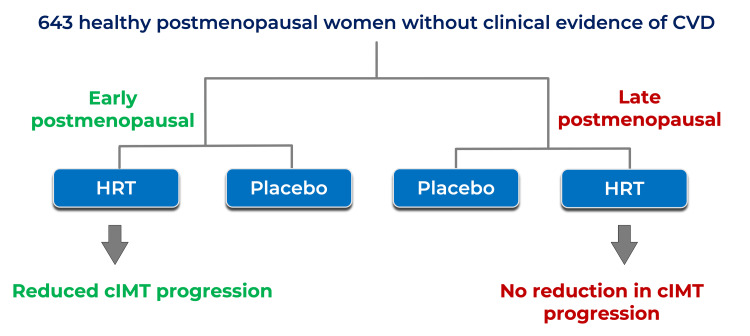
The ELITE cIMT, carotid intima-media thickness; HRT, hormone replacement therapy; CVD, cardiovascular disease; ELITE, Early Versus Late Intervention Trial With Estradiol Source: This image was created by Dr. Jomar Machuca.

The Timing Hypothesis is based on the healthy endothelium hypothesis, which describes estrogen’s dual effect on blood vessels. In healthy vasculature, estrogen inhibits the natural progression of atherosclerotic disease. However, the reduction in atherosclerosis progression is nonsignificant when plaque is already present in the arteries [5]. The Early Versus Late Intervention Trial With Estradiol (ELITE) published in 2016 tested the Timing Hypothesis by evaluating the effects of HRT on the progression of atherosclerosis in early postmenopausal women (i.e., less than six years from their last menses) compared to late postmenopausal women (i.e., more than 10 years from their last menses). CIMT was used to measure the progression of atherosclerosis. The study evidenced that, compared to placebo, HRT initiated early after menopause reduced the progression of subclinical atherosclerosis but had no effect when started late after menopause (Figure 2) [2,5,12-14]. This confirmed the Timing Hypothesis and illustrated the antiatherogenic effect of HRT on the healthy endothelium. 

Types of HRT and their effects

HRT has several systemic effects, including an increase in HDL and a reduction of LDL. It also increases triglycerides by up to 20%; however, this is not clinically significant. HRT also mediates increased coagulation factors and resistance to activated protein C, leading to a hypercoagulable state [8]. There are different types of estrogen therapy, including systemic estrogen therapy, which is effective for treating vasomotor symptoms and genitourinary syndrome and is also approved for osteoporosis prevention [13]. Conjugated oral equine estrogen in combination with bazedoxifene is approved for vasomotor symptoms and osteoporosis prevention in women with a uterus. This combination is an alternative for women without hysterectomy who do not tolerate progesterone [12,13].

Oral vs. Transdermal HRT

When comparing oral versus transdermal HRT, it should be noted that their effectiveness is similar. No clinical trials, however, compare the risk of thromboembolic events between oral and transdermal therapy [12-15]. Observational studies demonstrate that oral therapy is associated with a greater risk of thromboembolic events and increased triglyceride levels. In comparison, transdermal therapy has a neutral effect on triglyceride levels and a lower risk of developing hypertension [5,13]. Contrary to transdermal therapy, oral therapy is linked to increases in C-reactive protein [13]. Thus, as per observational studies, transdermal therapy has a more favorable side effect profile.

Vaginal Estrogen Therapy 

On the other hand, vaginal estrogen therapy is effective for genitourinary symptoms. While on this therapy, estrogen levels remain within postmenopausal physiological parameters, so it is an alternative for women with contraindications to systemic estrogen therapy (e.g., history of breast cancer or venous thromboembolism) [13,16].

Compounded HRT

It is important to note that there is limited data on the efficacy and safety of compounded HRT. It lacks a label describing its risks and is prone to contamination and dose variability. Therefore, it is not approved by the FDA [12,13].

HRT in patients with traditional risk factors for CVD

It is crucial to discuss the impact of hormone therapy in postmenopausal women with traditional comorbidities for CVD, such as obesity, metabolic syndrome, dyslipidemia, hypertension, and diabetes mellitus.

Obesity

Obesity itself predisposes to the development of thromboembolic events. Therefore, overweight and obese patients on hormonal therapy have a three to six times greater risk of venous thromboembolism, especially with the oral formulation. The liver's first-pass metabolism of oral estrogen increases the production of clotting factors, promoting a hypercoagulable state [13]. Similarly, patients with metabolic syndrome are also at increased risk of developing blood clots [5].

Hypercholesterolemia

Although HRT reduces cholesterol levels, this has not reduced cardiovascular events. Patients with LDL levels greater than or equal to 130 mg/dL receiving HRT are at increased risk of developing coronary artery disease [13].

Hypertension

The WHI study showed that hormone therapy can increase blood pressure by 1 to 1.5 mmHg, which may not seem significant. However, studies have shown that even a slight reduction of 1 mmHg in systolic blood pressure can result in a 2% decrease in the relative risk of major cardiovascular events and a 3% decrease in heart failure events [13]. Therefore, this should be taken into consideration when initiating HRT in patients with a history of hypertension.

Diabetes Mellitus

Diabetes is strongly associated with CVD, increasing it up to four times in women [13]. Studies show that HRT positively affects glycemic control and insulin resistance in postmenopausal women with and without diabetes. However, this has not resulted in reduced cardiovascular events, and recommendations for using HRT to prevent diabetes have not been established [13]. All these comorbidities should be addressed when starting HRT.

Indications of HRT

Various medical societies, such as the American College of Obstetricians and Gynecologists (ACOG), the North American Menopause Society, and the Endocrinology Society, concur that the primary indication of HRT is for treating menopausal symptoms, particularly vasomotor and genitourinary symptoms that significantly affect a woman's quality of life. Most of these societies suggest using the lowest effective dose, and the American College of Endocrinology specifies that therapy should not exceed five years [5]. It is important to note that although HRT has beneficial effects in reducing the progression of atherosclerosis in early menopausal women, it is not approved for the primary prevention of CVD [5].

Risks and contraindications of HRT

When it comes to the risks of HRT, numerous randomized controlled trials have demonstrated that these are uncommon when started in early menopausal women [5]. According to the WHI study, the use of hormonal therapy does not significantly increase the risk of pulmonary embolus, cerebrovascular infarction, breast cancer, diabetes, or total causes of death in women under 60 years of age. The only significant risk identified is the formation of deep vein thrombi secondary to the hypercoagulable state caused by HRT [5]. However, the decision to start HRT should be individualized by considering each patient's comorbidities and CVD risk. In general, HRT is not recommended for patients older than 60 years, with more than 10 years since menopause, or with an Atherosclerotic Cardiovascular Disease (ASCVD) risk of more than 10% [13-15]. Contraindications to systemic hormonal therapy include a history of thromboembolic events, breast or endometrial cancer, coronary artery disease, and a history of cerebrovascular infarction, among others [5,13].

Family History of Breast Cancer

Multiple studies have analyzed the use of HRT in women with a family history of breast cancer. For instance, a long-term follow-up of over 16,000 postmenopausal women in the WHI study found no significant association between having a first-degree relative with breast cancer and the development of breast cancer in postmenopausal women on HRT [17]. Another study also demonstrated no increase in the relative risk of breast cancer associated with HRT use and having a family history of breast cancer [18]. Similarly, a prospective cohort study of over 41,000 menopausal women showed that the rate of breast cancer development among women with a family history of breast cancer who received HRT for at least five years was similar to that of women who had never used HRT [19]. According to the British Menopause Society, HRT does not pose an additional risk for women with a family history of breast cancer [20].

## Conclusions

In closing, CVD is the number one cause of mortality in women. Postmenopausal women experience an acceleration of cardiovascular risk, resulting in a significantly higher number of cardiovascular events compared to premenopausal women. The Timing Hypothesis proposes that providing HRT at a critical moment in a woman's life can reduce the progression of atherosclerotic disease. This is explained by estrogen’s dual effect on the vasculature, limiting the progression of atherosclerosis only in patients with subclinical atherosclerotic disease. HRT can reduce overall mortality and has minimal side effects when started in women under 60 years of age and within the first 10 years after menopause. It is not approved for the primary prevention of CVD. Deciding to start HRT should be based on an individualized risk evaluation that takes into account the patient's age, time since the onset of menopause, and her comorbidities. In women suitable for HRT, it can be beneficial for treating menopausal symptoms to improve life quality. 
